# Setting research priorities for maternal, newborn and child health, sexual and reproductive health and nutrition in Afghanistan: an application of the Child Health and Nutrition Research Initiative methodology

**DOI:** 10.1136/bmjgh-2024-018579

**Published:** 2025-09-02

**Authors:** Tanvi Majumdar, Emily C Keats, Hana Tasic, Sama El Baz, David H Peters, Najibullah Safi, Hannah Tappis, Nadia Akseer, Afghanistan MNCH Research Prioritization Collaborators, Muhammad Sohail Afzal

**Affiliations:** 1 Department of International Health, Johns Hopkins University Bloomberg School of Public Health, Baltimore, Maryland, USA; 2 Modern Scientist Global, St. Catharines, Ontario, Canada; 3 School of Global Health, York University, Toronto, Ontario, Canada; 4 Independent Consultant, Kabul, Afghanistan; 5 Johns Hopkins Center for Humanitarian Health, Baltimore, Maryland, USA; 6 Johns Hopkins University, Baltimore, Maryland, USA

**Keywords:** Public Health, Maternal health, Health services research, Child health, Global Health

## Abstract

**Background:**

Since 2021, Afghanistan has faced a worsening humanitarian crisis that disproportionately impacts Afghan women and children. They experience inequities in healthcare access, deterioration of healthcare quality and extreme food insecurity. This study aims to fill an important gap by providing consensus on research priorities for maternal, newborn and child health (MNCH), sexual and reproductive health (SRH) and nutrition in Afghanistan.

**Methods:**

The Child Health and Nutrition Research Initiative (CHNRI) is a widely used research prioritisation methodology that crowdsources input from subject matter experts to generate, score and rank research questions. This study reached out to 303 Afghanistan health researchers, who were identified through relevant publications, to align on the 20 highest priority MNCH, SRH and nutrition research questions. Question generation occurred in 2022, and data collection and analysis were completed by January 2025.

**Results:**

The CHNRI exercise had 81 respondents, of which 53% were of Afghan origin. The 20 highest priority research questions for MNCH, SRH and nutrition in Afghanistan were mostly *description* and *delivery* questions in MNCH and nutrition topic areas. The top questions ranged from characterising the availability, access and quality of MNCH services, to leveraging locally available interventions for malnutrition and food security, to strategies for increasing immunisation coverage.

**Conclusion:**

By identifying high-priority research questions, donors, researchers, implementers and governments can align their research agendas and resource allocation to address critical health challenges for women and children in Afghanistan.

**WHAT IS ALREADY KNOWN ON THIS TOPIC**

- Since Afghanistan’s regime change in 2021, the health of women and children has been negatively impacted. A defined, collaborative research agenda is needed to effectively improve maternal, newborn and child health (MNCH), sexual and reproductive health (SRH) and nutrition for vulnerable groups in Afghanistan.

**WHAT THIS STUDY ADDS**

- This is the first application of the Child Health and Nutrition Research Initiative methodology to identify the 20 highest priority research questions for MNCH, SRH and nutrition in Afghanistan. The top 20 questions ranged in topic but were mostly *description* and *delivery* questions for MNCH and nutrition.

**HOW THIS STUDY MIGHT AFFECT RESEARCH PRACTICE OR POLICY**

- The results can align research agendas and resource allocation among donors, the de facto authorities, researchers and implementers to address vital MNCH, SRH and nutrition challenges in Afghanistan.

## Background

 Due to nearly three decades of political, social and economic instability compounded by natural disasters (eg, earthquake, flooding and drought), Afghanistan ranks 182 out of 193 countries in the United Nations’ Human Development Index Report for 2023–2024.^1^,^3^ Despite this low ranking, Afghanistan has improved in key health indicators, in part due to contributions from international development organisations and donors that funded and implemented health services in Afghanistan.^1^ Between 2003 and 2015, under-five mortality decreased by 29%, births in health facilities increased by 26% and childhood vaccination coverage rates doubled.^4^ From 2003 to 2018, the percentage of women having at least one antenatal care visit increased from 16% to 65.2%.^5^ From 2000 to 2020, the maternal mortality ratio (deaths per 100 000 live births) declined from 1346 to 620.^6^

However, after the regime change in 2021, the humanitarian crisis in Afghanistan has deepened and is eliminating significant gains. Today, women and girls have less access to healthcare, extreme poverty is widespread, international funding has significantly decreased and the healthcare system is near collapse.^1 2^ While much of the status of health in Afghanistan is unclear, it appears to be on a steep decline. It is projected that 14.8 million people in Afghanistan will face high acute food insecurity requiring urgent action in 2025 compared with 10.3 million people in 2020.^7 8^ A late 2021/early 2022 survey of female and male Afghan healthcare workers exposed the decreased availability of maternal and child healthcare, the deterioration of quality of care, a perceived increase in child malnutrition and increasing difficulties for females to access healthcare facilities and services.^9^ In 2024, an edict was issued banning women from attending public and private health facilities without a *mahram* (male companion), further limiting access to maternal, antenatal and neonatal care.^10^ Improving health in Afghanistan for women and children is critical to save lives and avoid further loss of health gains.

As we approach the 2030 benchmark for the United Nations’ Sustainable Development Goals, we must streamline and prioritise health research in Afghanistan to prevent further backsliding. To achieve this, we must first align researchers, implementers, the de facto authorities and donors on the highest priority health research questions for Afghanistan to efficiently direct resources and produce the greatest impacts.

This paper reports on the application of the Child Health and Nutrition Research Initiative (CHNRI) methodology to systematically, transparently and collaboratively set research priorities for maternal, newborn and child health (MNCH), sexual and reproductive health (SRH) and nutrition in Afghanistan. This is the first CHNRI exercise to focus on these topics in Afghanistan. They are particularly relevant as women and girls are disproportionately affected. Discrete CHNRIs have been conducted for other health topics in Afghanistan, including health systems and communicable diseases, and their results will be published separately. Findings from this CHNRI exercise will support health researchers, de facto authorities and donors in making informed decisions that will ultimately help to address the health crisis for vulnerable groups in Afghanistan.

## Methods

The CHNRI method is an adaptable, systematic, transparent and collaborative method that has been widely used for research priority setting in many fields. It engages experts in the field through a form of ‘crowdsourcing’ to collectively generate research questions and score them based on standardised criteria. The highest scoring questions represent research topics and domains to be prioritised for research and resource allocation.^11 12^

The standard CHNRI method has been described in detail in many papers.^11^,^15^ This study applied the standard methodology in four phases: (1) selecting a small expert working group to define CHNRI parameters and criteria, (2a) identifying a large group of experts to generate research questions and later score them based on the criteria, (2b) synthesising questions, developing the CHNRI survey and scoring questions and (3) calculating the Research Priority Scores (RPSs), expert agreement level and overall ranking of questions. A phase-by-phase explanation is below.

### Phase 1: establishing an expert working group and defining scope, context and evaluation criteria

A strategic advisory board (SAB) of experts on health in Afghanistan, MNCH and the CHNRI methodology was created to advise on the scope (short-term vs long-term research priorities), the context (health research priorities vs health programming priorities or both) and the criteria against which the questions would be scored for this CHNRI exercise. SAB members were identified through relevant publications and included Afghan and non-Afghan experts with a range of professional health experience. The list of eight SAB members and their credentials can be found in online supplemental Table 1.

To select the five criteria against which to score questions for this CHNRI, an email survey with an initial list of 13 relevant criteria was sent to the SAB. These criteria were based on other CHNRI exercises that noted criteria relevant for Afghanistan.^16^ The SAB ranked the top five criteria and the related criteria questions were developed (table 1). All five criteria were weighted equally in the analysis because all criteria were determined to be of equal importance by the study team and SAB.

**Table 1:** Criteria and criteria questions for the Afghanistan research prioritisation exercise

Criterion	Criteria questions
Feasibility	Is it feasible to conduct this research considering cultural, financial, human resources, logistical and security challenges?
Effectiveness	Would the intervention that arises from the proposed research be effective in improving health outcomes in Afghanistan?
Equity	Can answering the question facilitate interventions that reduce population inequities (ie, to improve the health of the most vulnerable and disadvantaged)?
Answerability	Would you say it is possible to design an ethically sound and implementable research study (eg, feasible, well-defined end points/outcomes, culturally appropriate) that can provide the requested answer?
Disease burden reduction	Would you say that interventions arising from the research would eventually contribute to a significant reduction in mortality/morbidity across the country?

### Phase 2A: identifying experts to generate and later score research questions that cover any health topic area in Afghanistan

#### Expert identification

To create a robust and sizeable group of experts to participate in the CHNRI, researcher mapping was conducted to identify researchers with three or more peer-reviewed publications on any health topic in Afghanistan from 2005 to 2022. PubMed and Scopus were used to search for researchers and to verify the number of articles published by each researcher. Keywords for search engines included “health AND Afghanistan” and “NOT soldiers, Iraq, military, army.” For identified researchers meeting the inclusion criteria, their contact information, affiliations, number of publications related to health in Afghanistan and areas of expertise were recorded in Excel. A total of 303 researchers were identified.

#### Research question generation

The 303 identified researchers were requested by email in 2022 to submit two to three highest priority, in their opinion, research questions related to any health topic area in Afghanistan. Submitted questions were recorded in Excel with the submitter’s information. A total of 96 researchers submitted 323 research questions across any health topic in Afghanistan.

Research questions were organised in Excel by topic area. Topic areas were health systems, communicable diseases, MNCH, SRH and nutrition. This CHNRI consisted of the MNCH, SRH and nutrition questions.

Note that we also conducted two other CHNRI exercises for research questions in the topic areas of: (1) health systems^17^ and (2) communicable diseases (forthcoming). All three CHNRI exercises originated from the same point (eg, the same SAB and the same 303 researchers) but diverged at the next phase. A separate survey was created for each CHNRI and sent to researchers at different time points. However, each CHNRI survey was sent to the same people who self-selected to respond.

### Phase 2B: synthesising research questions in MNCH, SRH and nutrition topic areas, survey development and research question scoring

#### Research question synthesis and survey creation

Question synthesis and survey creation for the MNCH, SRH and nutrition CHNRI were completed in 2024. Between 2022 and 2024, time was needed for preparatory work, and there were logistical constraints for the study team as team members changed. To synthesise MNCH, SRH and nutrition questions, the questions were carefully reviewed for clarity, redundancy and meaning. Through several rounds, the questions were synthesised and edited while ensuring fidelity to the researchers’ original question. For instance, repeat questions were condensed into a single question, questions with acronyms or unclear language were clarified, and multiple questions with similar content but different phrasing were combined when possible. An artificial intelligence tool, ChatGPT (V.3.5), was used to help with the process of combining questions. If two questions were similar, they were entered into ChatGPT, which was then prompted to synthesise the questions into a single question. The study team reviewed and edited the results to determine the finalised question, maintaining the original essence of the question.

Research questions were also categorised into the CHNRI 4D framework, in which the proposed research is related to domains of *description, delivery, development* or *discovery*.^16^ Table 2 displays definitions of each 4D domain.

**Table 2:** CHNRI 4D framework

Domain	Explanation
*Description*	‘Description’ research includes any proposed health research that would allow researchers to assess the burden of health problems in the population of interest and understand its determinants—that is, negative effects of risk factors and positive effects of delivered health interventions. This is typically achieved through epidemiological research.
*Delivery*	‘Delivery’ research includes all research questions that allow researchers to optimise health status of the population using the means that are already available. This is typically achieved through implementation research, operations research and/or health policy and systems research.
*Development*	‘Development’ research is focused on improving health interventions that already exist, but could be made more effective, affordable or sustainable.
*Discovery*	‘Discovery’ research includes all research questions that would lead to innovation, that is, generation of new knowledge to develop entirely new health interventions.

CHNRI, Child Health and Nutrition Research Initiative.

After synthesis, there were a total of 56 questions to score and rank in the survey for this CHNRI exercise. This survey consisted of 30 MNCH questions, nine SRH questions and 17 nutrition questions.

Johns Hopkins University’s Research Electronic Data capture (REDCap) was used to create the CHNRI survey to send to researchers. This survey would gather demographic information and then ask researchers to score the list of 56 MNCH, SRH and nutrition questions against the five criteria questions found in table 1. The survey was programmed to present all 56 questions in a random order for each respondent to avoid bias due to respondent fatigue.

#### Research question scoring

Using REDCap, the finalised survey was sent to all 303 identified Afghanistan health researchers, regardless of whether they had submitted research questions for these topic areas, in November 2024.

Researchers were given 4 weeks from November to December 2024 to complete the survey with weekly reminders sent through REDCap. Due to the busy end-of-year season, the survey response period was extended for 2 weeks in January 2025, with weekly reminders, to boost the response rate.

For each research question, respondents could answer, ‘Yes’ (1 point), ‘No’ (0 points), ‘Undecided’ (0.5 points) or ‘Insufficiently Informed’. Survey respondents could score all or some questions on the survey.

### Phase 3: calculating RPSs

In January 2025, after the close of data collection, the highest-scoring research questions were identified by calculating the RPS and the average expert agreement (AEA).

RPS is calculated as a simple mean. As previously mentioned, each respondent can assign each question a value of ‘Yes’ (1 point), ‘No’ (0 points), ‘Undecided’ (0.5 points) or ‘Insufficiently Informed’ for each of the five criteria. Responses of ‘Insufficiently Informed’ were excluded from RPS calculation. The points scored for each question are summed and averaged, resulting in an overall RPS score per question and a criterion-specific RPS score ranging from 0 to 1, which is equivalent to 0–100% per question. Research questions were ranked by RPS score. A higher overall RPS score indicates that the research question ranks higher in priority.

AEA is a measure of mode. It is the proportion of respondents selecting the most common score for each research question, measuring the level of agreement among respondents. AEA was calculated by dividing the number of respondents who gave the most common score by the total number of respondents answering that question. An AEA of 75% and above indicates a high level of agreement.

A sensitivity analysis was conducted to see how the question ranking differed if responses from partially completed surveys were excluded. The final question ranking was very similar when partially completed surveys were included or excluded. Both complete and partial survey responses were used in final data analyses.

The statistical software package R V.3.6.0 was used for all data analyses.

Questions were ranked overall (ie, across MNCH, SRH and nutrition) and within topic area (MNCH, SRH or nutrition).

### Patient and public involvement

The public (those with non-academic lived experiences within the Afghan community and/or in Afghanistan) was not involved in the research process due to significant logistical barriers, including restricted access to key populations such as women and girls in Afghanistan.

## Results

### Characteristics of respondents

Table 3 displays the demographic characteristics of the respondents. Out of the 303 invitees, 81 people responded and 59 respondents completed the entire survey (scored all 56 questions). About 53% (n=43) of all respondents were of Afghan origin. The response rate, including all respondents, was about 26.73% (n=81).

**Table 3:** Characteristics among all survey respondents

Characteristic	Proportion N(%)
Afghan Non-Afghan	43 (53.09) 38 (46.91)
High-income country Low or middle-income country	53 (65.43) 28 (34.57)
Years of experience in health research:	
1–3 years 4–7 years 8–10 years 11–15 years 16–20 years 20+ years	5 (6.17) 7 (8.64) 11 (13.58) 15 (18.52) 17 (20.99) 26 (32.10)
Years of health research experience related to Afghanistan:	
1–3 years 4–7 years 8–10 years 11–15 years 16–20 years 20+ years	18 (22.22) 16 (19.75) 16 (19.75) 12 (14.81) 10 (12.35) 9 (11.11)
Area of expertise (self-identified)*:	
Health systems Maternal and child health Communicable diseases Nutrition Sexual and reproductive health Mental health Non-communicable diseases Disabilities and injuries Others	55 (67.90) 52 (64.20) 32 (39.51) 21 (25.93) 22 (27.16) 11 (13.58) 15 (18.52) 1 (1.23) 12 (14.81)
Place of employment*:	
Academic institution Non-governmental organisation Healthcare setting Governmental agency Donor agency Other	48 (59.26) 20 (24.69) 9 (11.11) 4 (4.94) 1 (1.23) 11 (13.58)

* Respondents could have selected more than one option.

### Highest-scoring research questions

The 20 highest-scoring research questions based on overall RPS score are presented in table 4 (questions 1–10) and online supplemental file 1 (all ranked questions). The overall RPS score for the top 20 research questions ranged from 86.39% to 92.05%. The overall RPS score for all 56 scored research questions ranged from 54.20% to 92.05%.

**Table 4:** Overall ranking, research question, 4D’s domain, topic area, Intermediate Research Priority Scores (RPSs), Overall Research Priority Scores and average expert agreement for the top 10 research questions (based on highest overall RPS score)

Research question and ranking	Domain and topic area	Feasibility	Effectiveness	Equity	Answerability	Disease burden reduction	Overall RPS	AEA
**1**. What care is available for small or premature babies born in health facilities in Afghanistan?	Description (MNCH)	0.96	0.91	0.90	0.94	0.89	**92.05%**	**0.85**
**2**. What are potential interventions that are locally adaptable and scalable for preventing and managing the most common causes of child morbidity and mortality in Afghanistan, including acute respiratory infections, diarrhoeal illnesses and severe/moderate acute malnutrition?	Development (nutrition)	0.92	0.92	0.90	0.90	0.87	**90.15%**	**0.83**
**3**. What is the current availability, accessibility and quality of routine and emergency maternal, newborn and child health services in the public and private sector in Afghanistan, and what are the barriers to using these services when considering different socioeconomic strata (eg, region, urban/rural, etc)?	Description (MNCH)	0.91	0.92	0.90	0.88	0.88	**89.72%**	**0.83**
**4**. What are local sources of nutrition in Afghanistan to prevent child malnutrition and what are strategies to promote these sources?	Development (nutrition)	0.93	0.90	0.85	0.89	0.89	**89.27%**	**0.82**
**5**. In the context of Afghanistan’s sociocultural dynamics and fragile health system, what are strategies to achieve increased coverage of vaccines among women and children?	Development (MNCH)	0.87	0.88	0.89	0.88	0.93	**88.78%**	**0.81**
**6**. Postpartum haemorrhage is the leading cause of maternal death in Afghanistan. Thus, what are the feasible and sustainable options for prevention and management of postpartum haemorrhage in Afghanistan?	Development (MNCH)	0.86	0.89	0.93	0.85	0.90	**88.61%**	**0.82**
**7**. What are the root causes for stagnant PENTA3 (diphtheria, tetanus, pertussis, hepatitis B and Haemophilus influenzae type B) vaccination since 2013 in Afghanistan?	Description (MNCH)	0.90	0.87	0.87	0.90	0.87	**88.39%**	**0.84**
**8**. What is the association between maternal knowledge, attitudes and practices on adequate complementary feeding and malnutrition in children in Afghanistan?	Description (nutrition)	0.90	0.87	0.88	0.91	0.86	**88.21%**	**0.82**
**9**. What is the updated prevalence of micronutrient deficiencies (Vitamin A, iron, zinc, etc) among girls, women of reproductive age (15–49 years) and children under age 5 years at the national, province and district level in Afghanistan?	Description (nutrition)	0.81	0.92	0.92	0.89	0.87	**87.97%**	**0.80**
**10**. What are the options for managing high-risk pregnancies in extremely low resource regions of Afghanistan?	Delivery (MNCH)	0.87	0.86	0.90	0.88	0.86	**87.59%**	**0.77**

AEA, average expert agreement; MNCH, maternal, newborn and child health.

The AEA scores for the top 20 research questions ranged from 0.77 to 0.85, indicating a high level of overall agreement. In contrast, AEA scores for all 56 research questions varied from 0.40 to 0.85, indicating questions with low levels of overall agreement. Online supplemental Table 2 also shows a clear trend that questions with lower RPS scores had lower AEA scores.

**Figure 1:**
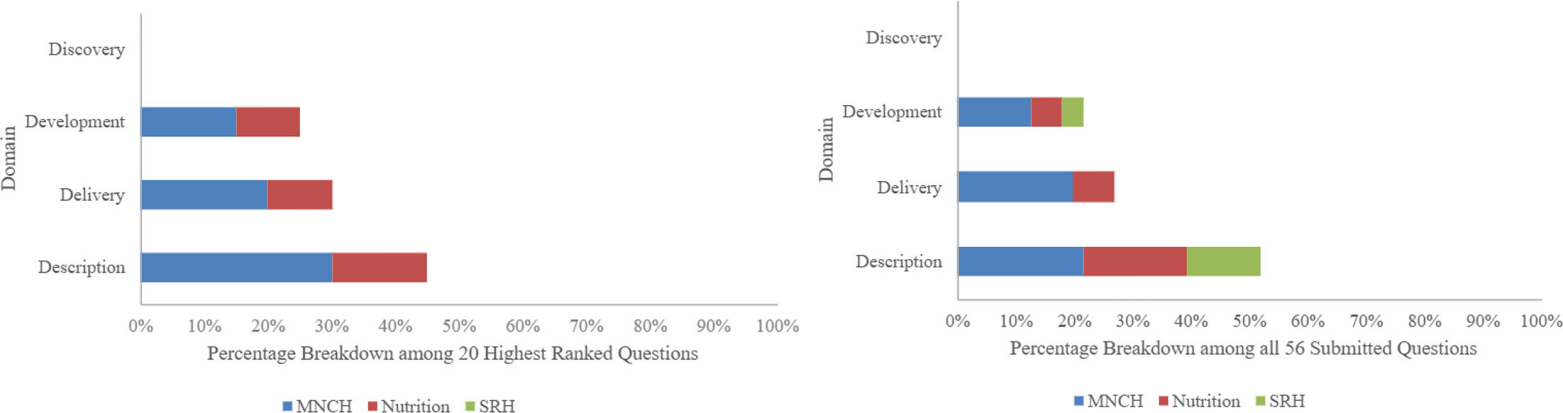
Percentage breakdown of questions across each topic area and domain among the top 20 highest ranked questions compared with all 56 questions submitted for ranking. MNCH, maternal, newborn and child health; SRH, sexual and reproductive health.

Figure 1 summarises the breakdown of the top 20 questions by domain and topic area, as compared with all 56 submitted questions. For the top 20 questions, 45% (n=9) of questions were in the *description* domain, 30% (n=6) were in the *delivery* domain and 25% (n=5) were in the *development* domain. The top 10 questions were *description* or *development*. None of the 56 questions scored were in the *discovery* domain. Among the top 20 questions, 65% (n=13) of questions fell into the MNCH topic area, 35% (n=7) of questions fell in the nutrition topic area and 0% (n=0) of questions were in the SRH topic area.

The percentage of MNCH, nutrition and SRH questions in the top 20 may reflect the breakdown of topic areas across the 56 questions scored. Among all 56 questions, there were 54% (n=30) MNCH questions, 30% (n=17) nutrition questions and only 16% (n=9) SRH questions. While most of the top 20 questions were *description*, and 78% (n=7) of all scored SRH questions were *description*, no SRH questions appeared in the top 20. The ranking of all questions separated by topic area is shown in online supplemental Tables 3-5.

The top 20 research questions covered issues including characterising the availability, access and quality of MNCH services, leveraging locally available nutrition interventions for children and food security, strategies to increase immunisation coverage, combatting causes of maternal death and poor birth outcomes, prevalence of nutrient deficiencies, community engagement strategies to improve MNCH and nutrition and characterising current MNCH morbidity and mortality. The top three research questions were two MNCH *description* questions and one nutrition *development* question. These MNCH questions related to the care available for premature babies and a general understanding of the availability, accessibility and quality of MNCH services and the barriers to accessing them. The nutrition question focused on locally adaptable interventions to prevent and manage causes of child morbidity, including malnutrition.

## Discussion

To the best of our knowledge, this is the first CHNRI application focused on MNCH, SRH and nutrition in Afghanistan. The response rate (26.73%) was in line with other CHNRI applications that cited response rates from 15% to 35% for the final question scoring phase.^18^ The 20 highest priority research questions were mostly *description* and *delivery-related* questions in MNCH and nutrition topic areas. The top questions ranged from characterising the availability, access and quality of MNCH services to leveraging locally available interventions for malnutrition and food security to strategies for increasing immunisation coverage.

The high proportion of *description* questions among the top 20 questions presents a need to assess the burden of and access to care for MNCH and nutrition issues in Afghanistan. Pregnant women in rural areas have reported extreme difficulties in reaching healthcare workers.^19^ The 2024 ban on women attending public and private medical institutes can prevent women from becoming medical professionals, potentially creating a shortage of female healthcare workers.^10^ In some rural provinces, new decrees prevent men from providing healthcare to women outside of emergencies.^9^ If there is a shortage of female healthcare providers, women’s access to MNCH and SRH services will be further jeopardised. Additionally, accurate health surveillance may be a challenge in Afghanistan as 90% of healthcare facilities are at risk of closure due to instability, shortage of medicines and failure to pay healthcare workers.^20^ A 2016 study noted that low-quality recordkeeping for maternal morbidities and mortality is a major issue in Afghanistan.^21^ Between low access to care and inconsistent records, *description*-related issues are major challenges. Prioritising research on these topics can help identify gaps in services needed to reach vulnerable groups.

With many of the top 20 questions being *delivery* questions, there is an indication that improved implementation of existing health interventions is plausible and effective, given Afghanistan’s resource constraints. For example, one *delivery* question was related to using community-based interventions to improve nutrition. A similar programme was previously implemented in Afghanistan and found that illiterate women were able to use nutrition strategies to positively impact children’s weight.^22^ As the 2022–2023 Multiple Indicator Cluster Survey reported 44.7% of children were stunted, delivery of nutrition interventions is a high priority.^1 23^ Research on these topics could help close gaps in accessing care by increasing or improving the delivery of existing interventions.

*Development* questions appeared in the top 10 questions, further emphasising the need to improve existing interventions. For example, a top five *development* question asked about strategies to increase vaccination coverage considering Afghanistan’s sociocultural dynamics and fragile health system. Afghanistan struggles with disease control due to lack of medical supplies, health facility closures and low access to healthcare. Only 37% of children aged 12–23 months have full basic vaccinations, and Médecins Sans Frontières has reported a surge in endemic measles.^23 24^

The top 20 questions focused on MNCH and nutrition. This is in line with evidence of a high maternal mortality rate in Afghanistan, increasing limitations on access to maternal, antenatal and neonatal healthcare and increasing food insecurity.^7 10 20 23^ There were no SRH questions in the top 20, potentially due to their lower feasibility scores. With fewer women receiving education and medical training and the ‘taboo’ nature of SRH questions, SRH research may not be highly feasible. There were also no *discovery* questions, which reflect a greater need to address urgent health concerns with available resources considering Afghanistan’s instability, social complexities and loss of international aid.^1^ The lack of *discovery* questions is also consistent with the results from a 2025 Afghanistan CHNRI about health system research priorities.^17^

The findings from this CHNRI aligned with findings from other CHNRIs related to MNCH and nutrition in low- and middle-income countries (LMICs), but it differed in aspects that reflect the context-specific nature of health challenges and research priorities. A child wasting CHNRI found that the top priority questions were majority *description*, and the other top questions were related to *delivery* and *development* of existing interventions using available resources.^14^ A childhood nutrition CHNRI in Africa showed that half of the top priority questions were related to *delivery* strategies and the other half were related to improving existing interventions.^25^ A review of the first 50 applications of the CHNRI method showed that the results of LMIC CHNRIs tend to prioritise *delivery* questions to improve implementation and address urgent health issues with short time frames and greater consequences.^12^ This likely keeps LMICs from prioritising *development* and *discovery* research.^12^ An MNCH CHNRI in India also showed that *delivery* questions ranked highest in priority, though the India CHNRI used different scoring criteria than this Afghanistan CHNRI. Some of the same health issues appeared in the India CHNRI, including vaccination coverage and surveillance of infectious diseases and morbidities.^26^

These findings provide a guide for researchers, programme implementers, de facto authorities and donors to allocate resources and advocate for high-priority MNCH, SRH and nutrition issues in Afghanistan, especially considering resource limitations. These findings can streamline research proposal generation and acceptance. In addition to existing fluctuations and limitations in funding for research in Afghanistan, the 2025 change in the US presidential administration resulted in a dramatic reduction in the US’s financial contribution to global aid and development. Historically, the US has been the largest single-country donor. It is critical that other donors converge on an evidence-based agenda for MNCH, SRH and nutrition research in Afghanistan.

The 2025 Nutrition for Growth Summit resulted in a US$28 billion commitment for global nutrition development, particularly in LMICs with significant malnutrition, such as Afghanistan.^27^ This funding is promising for advancing a nutrition research agenda in Afghanistan, which can be guided by the findings of this study.

Additionally, this application of the CHNRI methodology adds to the body of evidence that it is a useful tool that can be adapted to many fields.

Ultimately, the context around conducting research in Afghanistan is highly complex. The high-priority research areas found in this CHNRI may be challenging to research in practice due to funding constraints, pullbacks from implementing organisations and barriers to access through the de facto authorities. Implementation and policy-related challenges have thrown the current research agenda in Afghanistan into greater flux. However, as found in this CHNRI, there is need for research with a gender focus because the restrictions on women and girls are likely to affect their access to high-quality healthcare, resulting in grave implications for mortality and morbidity among women, children and infants. Addressing high-priority and gender-oriented research topics will improve researchers’ abilities to drive effective changes in healthcare delivery, infrastructure and policies in Afghanistan. Without advocacy for health research in Afghanistan, there is great risk of missing critical health information or implementing interventions in less effective and efficient ways.

### Limitations

The limitations of this study include respondent coverage, selection bias, question synthesis and the timing of data collection.

While 303 researchers were invited to participate, only 81 researchers participated in the CHNRI exercise. Participants were invited to participate based on academic publications for logistical feasibility, and most participants came from academic institutions or non-governmental organisations. There was no inclusion of unpublished researchers/people and limited perspectives from other places of employment. Stakeholders within Afghanistan’s government and communities were not included due to logistical challenges. Unrepresented stakeholders may have scored questions differently, especially for the criteria ‘feasibility’ and ‘answerability.’

CHNRI participants self-selected to respond to the survey, and there may be inherent differences between responders and non-responders. Respondents came from several areas of expertise, so individuals may have scored questions in certain topic areas differently based on their primary area of expertise.

Additionally, participants self-selected to generate questions for the CHNRI exercise. There may be other important MNCH, SRH or nutrition questions that were not included because of differences between responders and non-responders during question generation. Respondents provided many questions that the study team synthesised for clarity and conciseness to create a survey that could be realistically completed. It is possible that, during synthesis, some questions were removed if unclear or merged. Every effort was made to maintain the essence of each question.

Notably, the final survey length (56 questions) may have been too long for some participants to complete or it may have introduced bias due to respondent fatigue. Other CHNRIs should consider minimising survey length if the essence of the questions can be maintained.

Furthermore, questions were initially submitted for this CHNRI in 2022. Questions were open to revision until January 2025 when data collection was completed. While Afghanistan still struggles with many of the same issues today, there have been additional restrictions since the initial question generation period. For instance, the new ban on women studying medicine has implications for question generation and rankings.

Finally, this CHNRI exercise was completed right before the 2025 change in the US presidential administration, which resulted in massive shifts across the global aid and development sector. This administration has pulled back from funding and implementing major development and research programmes such as those funded through USAID, the Centers for Disease Control and Prevention and the National Institutes for Health. This change has significant implications for health questions, top research priorities and the future of health in Afghanistan.

## Conclusion

This application of the CHNRI method leveraged the expertise of Afghanistan health researchers to systematically, transparently and collaboratively set the top research priorities for MNCH, SRH and nutrition in Afghanistan. The highest priority research topics varied, including topics such as characterising the availability, access and quality of MNCH services, leveraging locally available interventions for malnutrition and food security and developing strategies to increase immunisation coverage. By identifying high-priority research questions, researchers, donors, implementers and the de facto authorities can align their approaches to address health challenges for vulnerable women and children in Afghanistan. Using the findings from this CHNRI as a roadmap, we can make progress towards the United Nations’ development goals for 2030, improving Afghanistan’s human development index and preventing diseases and avoidable loss of life.

## Supplementary material

10.1136/bmjgh-2024-018579 online supplemental file 1

## Data Availability

Data are available upon reasonable request.
