# Supplementary material for: Setting research priorities for maternal, newborn and child health, sexual and reproductive health and nutrition in Afghanistan: an application of the Child Health and Nutrition Research Initiative methodology
Source: BMJ Glob Health. 2025 Sep 2;10(Suppl 3):e018579. doi: 10.1136/bmjgh-2024-018579 (PMC12406864; doi:10.1136/bmjgh-2024-018579)
Supplement: online supplemental file 1 [file bmjgh-10-Suppl_3-s001.docx]

**Supplemental Document**

**Table of Contents**

[**Table 1.** Afghanistan CHNRI Strategic Advisory Board Members. 2](#_Toc204449389)

[**Table 2.** Overall Ranking, Research Question, 4D’s Domain, Topic Area, Intermediate Research Priority Scores, Overall Research Priority Scores, and Average Expert Agreement for all scored research questions. 4](#_Toc204449390)

[**Table 3.** Overall Ranking, Research Question, 4D’s Domain, Intermediate Research Priority Scores, Overall Research Priority Scores, and Average Expert Agreement for all MNCH research questions. 18](#_Toc204449391)

[**Table 4.** Overall Ranking, Research Question, 4D’s Domain, Intermediate Research Priority Scores, Overall Research Priority Scores, and Average Expert Agreement for all SRH research questions. 24](#_Toc204449392)

[**Table 5.** Overall Ranking, Research Question, 4D’s Domain, Intermediate Research Priority Scores, Overall Research Priority Scores, and Average Expert Agreement for all nutrition research questions. 25](#_Toc204449393)

##

## **Table 1.** Afghanistan CHNRI Strategic Advisory Board Members.

| **SAB Member Name** | **Organization** | **Position Title** | **Health Topic Expertise** | **Number of Afghanistan Publications** |
| --- | --- | --- | --- | --- |
| Catherine Todd, MD, MPH | Pact | Senior Technical Advisor, Global Health | MNCH, HIV, SRH, mHealth | 45 |
| Hannah Tappis, MPH, DrPH | Jhpiego  Johns Hopkins University Bloomberg School of Public Health | Principal Technical Advisor   Associate Faculty, Center for Humanitarian Health, Department of International Health | MNCH, Health Systems | 23 |
| David H. Peters, MD, DrPH | York University | Interim Provost | MNCH, Health Systems | 21 |
| Najibullah Safi, MD, MSc | World Health Organization | Program Manager, Health System Development | Health Systems, SRH, MNCH, Infectious Disease (COVID, Malaria) | 18 |
| Ahmad Shah Salehi, MD, MBA, MSc, PhD | Lapis Communications | Senior Public Health Advisor | MNCH, Health Systems | 12 |
| Kerri Wazny, PhD | Children’s Investment Fund Foundation | EME Manager - Nutrition | CHNRI Methodology | - |
| Mickey Chopra, MD, MPH, PhD | World Bank | Global Solutions Lead for Service Delivery | MNCH, Health Systems, Mental Health | 4 |
| Robert Black, MD, MPH | Johns Hopkins University Bloomberg School of Public Health | Director for the Institute of International Programs  Professor | Health Systems, MNCH, Nutrition  CHNRI methodology | - |

##

##

##

##

##

## **Table 2.** Overall Ranking, Research Question, 4D’s Domain, Topic Area, Intermediate Research Priority Scores, Overall Research Priority Scores, and Average Expert Agreement for all scored research questions.

| **Ranking** | **Research Question** | **Domain** | **Topic Area** | **Feasibility** | **Effectiveness** | **Equity** | **Answerability** | **Disease Burden Reduction** | **Overall RPS** | **AEA** |
| --- | --- | --- | --- | --- | --- | --- | --- | --- | --- | --- |
| 1 | What care is available for small or premature babies born in health facilities in Afghanistan? | Description | MNCH | 0.96 | 0.91 | 0.90 | 0.94 | 0.89 | **92.05%** | **0.85** |
| 2 | What are potential interventions that are locally adaptable and scalable for preventing and managing the most common causes of child morbidity and mortality in Afghanistan, including acute respiratory infections, diarrheal illnesses, and severe/moderate acute malnutrition? | Development | Nutrition | 0.92 | 0.92 | 0.90 | 0.90 | 0.87 | **90.15%** | **0.83** |
| 3 | What is the current availability, accessibility and quality of routine and emergency maternal, newborn and child health services in the public and private sector in Afghanistan, and what are the barriers to using these services when considering different socioeconomic strata (e.g., region, urban/rural, etc)? | Description | MNCH | 0.91 | 0.92 | 0.90 | 0.88 | 0.88 | **89.72%** | **0.83** |
| 4 | What are local sources of nutrition in Afghanistan to prevent child malnutrition and what are strategies to promote these sources? | Development | Nutrition | 0.93 | 0.90 | 0.85 | 0.89 | 0.89 | **89.27%** | **0.82** |
| 5 | In the context of Afghanistan's socio-cultural dynamics and fragile health system, what are strategies to achieve increased coverage of vaccines among women and children? | Development | MNCH | 0.87 | 0.88 | 0.89 | 0.88 | 0.93 | **88.78%** | **0.81** |
| 6 | Postpartum hemorrhage is the leading cause of maternal death in Afghanistan. Thus, what are the feasible and sustainable options for prevention and management of postpartum hemorrhage in Afghanistan? | Development | MNCH | 0.86 | 0.89 | 0.93 | 0.85 | 0.90 | **88.61%** | **0.82** |
| 7 | What are the root causes for stagnant PENTA3 (diphtheria, tetanus, pertussis, hepatitis B, and Haemophilus influenzae type B) vaccination since 2013 in Afghanistan? | Description | MNCH | 0.90 | 0.87 | 0.87 | 0.90 | 0.87 | **88.39%** | **0.84** |
| 8 | What is the association between maternal knowledge, attitudes, and practices on adequate complementary feeding and malnutrition in children in Afghanistan? | Description | Nutrition | 0.90 | 0.87 | 0.88 | 0.91 | 0.86 | **88.21%** | **0.82** |
| 9 | What is the updated prevalence of micronutrient deficiencies (Vitamin A, iron, zinc, etc) among girls, women of reproductive age (15-49 years) and children under age 5 years at the national, province and district level in Afghanistan? | Description | Nutrition | 0.81 | 0.92 | 0.92 | 0.89 | 0.87 | **87.97%** | **0.80** |
| 10 | What are options for managing high risk pregnancies in extremely low resource regions of Afghanistan? | Delivery | MNCH | 0.87 | 0.86 | 0.90 | 0.88 | 0.86 | **87.59%** | **0.77** |
| 11 | What are the most effective communication strategies for increasing awareness of Maternal, Infant and Young Child Nutrition (MIYCN) among the Afghan population? | Delivery | Nutrition | 0.90 | 0.87 | 0.87 | 0.90 | 0.84 | **87.41%** | **0.80** |
| 12 | How have bottom-up community engagement interventions impacted maternal, newborn and child health service utilization in Afghanistan and what is the need for community engagement interventions? | Delivery | MNCH | 0.87 | 0.88 | 0.89 | 0.85 | 0.88 | **87.37%** | **0.78** |
| 13 | What is the impact of community-level interventions for improving nutrition outcomes, including community-based growth monitoring and promotion and community level management of acute malnutrition? | Delivery | Nutrition | 0.84 | 0.91 | 0.88 | 0.86 | 0.87 | **87.35%** | **0.78** |
| 14 | What is the role of malnutrition in the health crisis women and children in Afghanistan are currently facing, and what are feasible, effective and scalable strategies for integrated MNCH and nutrition services in Afghanistan (e.g. management of mothers and infants at risk of moderate to severe malnutrition)? | Description | Nutrition | 0.88 | 0.90 | 0.87 | 0.86 | 0.85 | **87.22%** | **0.78** |
| 15 | What are the key factors (e.g., facility readiness, staffing) in improving quality of intrapartum care and monitoring in labour to decrease intrapartum stillbirth in Afghanistan? | Development | MNCH | 0.84 | 0.88 | 0.84 | 0.90 | 0.90 | **87.09%** | **0.81** |
| 16 | The three delays in maternal, newborn and child health service provision include (1) delays in access to quality maternal health care services (2) delays in availability of quality maternal health services and (3) delays in decision-making to utilize maternal health services in Afghanistan. What is the association between the three delays and maternal mortality in Afghanistan? | Description | MNCH | 0.86 | 0.91 | 0.86 | 0.88 | 0.85 | **87.07%** | **0.80** |
| 17 | Considering the low immunization coverage rates among Afghan children, what are immediate and cost-effective approaches to reducing child mortality due to vaccine-preventable diseases, compared to the current immunization strategies? | Delivery | MNCH | 0.92 | 0.85 | 0.88 | 0.85 | 0.85 | **86.86%** | **0.79** |
| 18 | What is the status of coverage, access, equity, demand and acceptability for immunization of children in Afghanistan after the regime change in August 2021? | Description | MNCH | 0.87 | 0.88 | 0.88 | 0.85 | 0.84 | **86.53%** | **0.77** |
| 19 | What is the current state of maternal, newborn, and child morbidity and mortality in Afghanistan, especially in rural areas? | Description | MNCH | 0.85 | 0.87 | 0.87 | 0.89 | 0.84 | **86.39%** | **0.80** |
| 20 | What are current feasible and sustainable options for maintaining and improving quality, accessibility and equity in maternal, newborn and child health service provision, especially in rural areas of Afghanistan? | Delivery | MNCH | 0.84 | 0.83 | 0.90 | 0.91 | 0.84 | **86.39%** | **0.80** |
| 21 | What nutritional interventions and resources are currently in place to address iron-deficiency anemia among women of childbearing age in Afghanistan and to what extent are these initiatives addressing the issue? | Delivery | Nutrition | 0.88 | 0.87 | 0.86 | 0.90 | 0.81 | **86.19%** | **0.77** |
| 22 | What is the role of midwives in supporting perinatal mental health? | Delivery | MNCH | 0.88 | 0.84 | 0.85 | 0.89 | 0.83 | **85.69%** | **0.76** |
| 23 | What strategies can be employed to identify vulnerabilities in food and nutrition security and effectively respond and provide emergency food assistance in Afghanistan? | Delivery | Nutrition | 0.90 | 0.86 | 0.85 | 0.84 | 0.83 | **85.63%** | **0.76** |
| 24 | What intervention models can be used to improve equity in maternal and child health and nutrition in light of vast discrepancies in stunting prevalence and vaccination coverage in different provinces? | Delivery | MNCH | 0.86 | 0.85 | 0.86 | 0.86 | 0.84 | **85.54%** | **0.75** |
| 25 | What evidence-based maternal health interventions can be implemented for women who cannot access a health facility? | Development | MNCH | 0.81 | 0.87 | 0.88 | 0.84 | 0.88 | **85.52%** | **0.78** |
| 26 | What are feasible, adaptable and sustainable strategies for conducting and improving mortality reporting and surveillance at national and sub-national levels for maternal, perinatal, stillbirth, newborn and child deaths in Afghanistan? | Delivery | MNCH | 0.86 | 0.84 | 0.88 | 0.86 | 0.84 | **85.39%** | **0.76** |
| 27 | What is the nutritional status (including stunting, wasting, and underweight) of children under the age of five in Afghanistan after the regime change? | Description | Nutrition | 0.84 | 0.84 | 0.88 | 0.87 | 0.84 | **85.21%** | **0.78** |
| 28 | What are the prevailing infant and young child feeding (IYCF) practices among children under 24 months in Badakhshan province, Afghanistan, and what insights can be drawn regarding the effectiveness of current nutrition interventions and recommendations for new interventions and scale-up strategies in the region? | Description | Nutrition | 0.89 | 0.83 | 0.85 | 0.90 | 0.77 | **84.85%** | **0.75** |
| 29 | What are feasible, effective and scalable strategies for integration of mental health and psychosocial support in MNCH programs? | Delivery | MNCH | 0.82 | 0.87 | 0.87 | 0.84 | 0.83 | **84.60%** | **0.74** |
| 30 | What is the current maternal mortality ratio and what are the primary contributors to maternal mortality in Afghanistan? | Description | MNCH | 0.80 | 0.84 | 0.88 | 0.86 | 0.85 | **84.45%** | **0.77** |
| 31 | What is the role of non-governmental organizations and civil society organizations in facilitating and delivering women's basic health services in the context of severe social restrictions? | Delivery | MNCH | 0.88 | 0.79 | 0.84 | 0.87 | 0.78 | **83.09%** | **0.75** |
| 32 | What is the prevalence of anemia among school children in Afghanistan, and how does it affect their academic performance? | Description | Nutrition | 0.79 | 0.83 | 0.85 | 0.84 | 0.82 | **82.55%** | **0.77** |
| 33 | What are feasible demand-side and supply-side options for promoting adequate birth spacing in Afghanistan? | Development | SRH | 0.74 | 0.85 | 0.84 | 0.87 | 0.83 | **82.49%** | **0.70** |
| 34 | What health services do women, including pregnant women, adolescent girls, children, and other vulnerable populations use, need and want, at national and subnational levels in Afghanistan? | Description | MNCH | 0.81 | 0.79 | 0.83 | 0.83 | 0.83 | **81.68%** | **0.72** |
| 35 | How can maternal, newborn and child health be improved in a context where policies have severely limited girls' and women's education? | Development | MNCH | 0.78 | 0.82 | 0.88 | 0.78 | 0.83 | **81.68%** | **0.72** |
| 36 | What are feasible solutions for meeting the critical health needs for women, including pregnant women, adolescent girls, children, and other vulnerable populations at national and subnational levels in Afghanistan? | Development | MNCH | 0.76 | 0.81 | 0.84 | 0.81 | 0.82 | **80.81%** | **0.69** |
| 37 | What are the prevalence and associated risk factors for anemia among pregnant women and women of childbearing age in Kandahar City as compared to Afghanistan overall? | Description | Nutrition | 0.79 | 0.83 | 0.77 | 0.87 | 0.78 | **80.81%** | **0.74** |
| 38 | What is the effectiveness of measles vaccination, including the seroconversion rate of measles-containing-vaccine first dose (MCV1) and measles-containing-vaccine second dose (MCV2) in Afghanistan? | Description | MNCH | 0.80 | 0.84 | 0.75 | 0.81 | 0.79 | **79.77%** | **0.72** |
| 39 | How can child immunization services in Afghanistan be sustained after GAVI funding (for equitable and sustainable vaccination of children in low- and middle-income countries) is phased out? | Delivery | MNCH | 0.83 | 0.80 | 0.78 | 0.78 | 0.80 | **79.64%** | **0.68** |
| 40 | What are the perceptions of men in Afghanistan toward family planning methods and what factors predict their contraception usage? | Description | SRH | 0.79 | 0.82 | 0.81 | 0.79 | 0.77 | **79.64%** | **0.68** |
| 41 | What are immediate actions that would maintain the gains achieved in maternal and child health outcomes in Afghanistan in the past decade? | Development | MNCH | 0.78 | 0.81 | 0.81 | 0.78 | 0.78 | **79.25%** | **0.68** |
| 42 | What is the extent of women's access to sexual and reproductive health services in Afghanistan, including maternity and well woman and family planning services; and how does this access vary among women of reproductive age living in urban, peri-urban, and rural areas of the country? | Description | SRH | 0.75 | 0.84 | 0.83 | 0.76 | 0.77 | **79.05%** | **0.70** |
| 43 | What are possible school-based nutrition programme types/models that can reduce adolescent malnutrition, and school dropout in Afghanistan? | Development | Nutrition | 0.78 | 0.79 | 0.78 | 0.82 | 0.75 | **78.38%** | **0.70** |
| 44 | What are the ways in which children's health can be appropriately prioritized in Afghanistan? | Delivery | MNCH | 0.79 | 0.78 | 0.77 | 0.78 | 0.78 | **77.86%** | **0.70** |
| 45 | How does the current state of family planning services in Afghanistan impact maternal, newborn, child, and reproductive health in the country? | Description | SRH | 0.70 | 0.77 | 0.84 | 0.78 | 0.77 | **77.30%** | **0.64** |
| 46 | What is the disease burden of reproductive tract infections among women in Afghanistan? | Description | SRH | 0.73 | 0.79 | 0.83 | 0.76 | 0.73 | **76.73%** | **0.64** |
| 47 | How has the regime-change in August 2021 impacted maternal, newborn and child mortality and burden of disease, as well as health service (e.g., antenatal care, postnatal care, skilled birth attendance) provision, access and utilization nationally and subnationally in Afghanistan? | Description | MNCH | 0.73 | 0.76 | 0.82 | 0.75 | 0.75 | **76.37%** | **0.65** |
| 48 | What is the status of menstrual hygiene management among women and girls in Afghanistan? | Description | SRH | 0.69 | 0.81 | 0.84 | 0.75 | 0.70 | **75.69%** | **0.63** |
| 49 | How do facility-based maternal and newborn mortality estimates compare to community-based mortality estimates, when considering the proportion of home birth and attendants, within and across districts in Afghanistan? | Description | MNCH | 0.75 | 0.75 | 0.76 | 0.79 | 0.72 | **75.37%** | **0.68** |
| 50 | What are the ethical implications of studying maternal, newborn and child health in a context of severe social restrictions on girls and women? | Description | MNCH | 0.66 | 0.71 | 0.80 | 0.76 | 0.71 | **72.93%** | **0.59** |
| 51 | Is there a significant impact of Vitamin D levels on the growth of children residing in apartment-style housing in Afghanistan? | Description | Nutrition | 0.77 | 0.73 | 0.74 | 0.73 | 0.63 | **72.14%** | **0.62** |
| 52 | Several barriers to contraception uptake have been identified in the literature. What are the current factors that have led to the stagnation of the modern contraceptive prevalence rate (mCPR) in Afghanistan since 2010, despite the country being positioned for rapid growth of mCPR, and what are the possible interactions and magnitudes of effect of the identified barriers to contraception uptake? | Description | SRH | 0.59 | 0.75 | 0.76 | 0.74 | 0.69 | **70.41%** | **0.57** |
| 53 | Following the regime change, how has the food insecurity situation affected child marriage in Afghanistan? | Description | Nutrition | 0.59 | 0.62 | 0.73 | 0.68 | 0.63 | **65.05%** | **0.52** |
| 54 | What are acceptable options for reducing child marriage and promoting contraceptive use in Afghanistan under the current regime? | Development | SRH | 0.40 | 0.74 | 0.72 | 0.65 | 0.74 | **65.05%** | **0.59** |
| 55 | What are common causes and contributing factors to infertility in Afghanistan? | Description | SRH | 0.55 | 0.63 | 0.61 | 0.61 | 0.52 | **58.11%** | **0.45** |
| 56 | How has the freezing of $9 billion foreign reserves after the regime change impacted the current health/malnutrition crisis for women and children in Afghanistan? | Description | Nutrition | 0.56 | 0.52 | 0.55 | 0.58 | 0.51 | **54.20%** | **0.40** |

## **Table 3.** Overall Ranking, Research Question, 4D’s Domain, Intermediate Research Priority Scores, Overall Research Priority Scores, and Average Expert Agreement for all MNCH research questions.

| **Ranking** | **Research Question** | **Domain** | **Feasibility** | **Effectiveness** | **Equity** | **Answerability** | **Disease Burden Reduction** | **Overall RPS** | **AEA** |
| --- | --- | --- | --- | --- | --- | --- | --- | --- | --- |
| 1 | What care is available for small or premature babies born in health facilities in Afghanistan? | Description | 0.96 | 0.91 | 0.90 | 0.94 | 0.89 | **92.05%** | **0.85** |
| 2 | What is the current availability, accessibility and quality of routine and emergency maternal, newborn and child health services in the public and private sector in Afghanistan, and what are the barriers to using these services when considering different socioeconomic strata (e.g., region, urban/rural, etc)? | Description | 0.91 | 0.92 | 0.90 | 0.88 | 0.88 | **89.72%** | **0.83** |
| 3 | In the context of Afghanistan's socio-cultural dynamics and fragile health system, what are strategies to achieve increased coverage of vaccines among women and children? | Development | 0.87 | 0.88 | 0.89 | 0.88 | 0.93 | **88.78%** | **0.81** |
| 4 | Postpartum hemorrhage is the leading cause of maternal death in Afghanistan. Thus, what are the feasible and sustainable options for prevention and management of postpartum hemorrhage in Afghanistan? | Development | 0.86 | 0.89 | 0.93 | 0.85 | 0.90 | **88.61%** | **0.82** |
| 5 | What are the root causes for stagnant PENTA3 (diphtheria, tetanus, pertussis, hepatitis B, and Haemophilus influenzae type B) vaccination since 2013 in Afghanistan? | Description | 0.90 | 0.87 | 0.87 | 0.90 | 0.87 | **88.39%** | **0.84** |
| 6 | What are options for managing high risk pregnancies in extremely low resource regions of Afghanistan? | Delivery | 0.87 | 0.86 | 0.90 | 0.88 | 0.86 | **87.59%** | **0.77** |
| 7 | How have bottom-up community engagement interventions impacted maternal, newborn and child health service utilization in Afghanistan and what is the need for community engagement interventions? | Delivery | 0.87 | 0.88 | 0.89 | 0.85 | 0.88 | **87.37%** | **0.78** |
| 8 | What are the key factors (e.g., facility readiness, staffing) in improving quality of intrapartum care and monitoring in labour to decrease intrapartum stillbirth in Afghanistan? | Development | 0.84 | 0.88 | 0.84 | 0.90 | 0.90 | **87.09%** | **0.81** |
| 9 | The three delays in maternal, newborn and child health service provision include (1) delays in access to quality maternal health care services (2) delays in availability of quality maternal health services and (3) delays in decision-making to utilize maternal health services in Afghanistan. What is the association between the three delays and maternal mortality in Afghanistan? | Description | 0.86 | 0.91 | 0.86 | 0.88 | 0.85 | **87.07%** | **0.80** |
| 10 | Considering the low immunization coverage rates among Afghan children, what are immediate and cost-effective approaches to reducing child mortality due to vaccine-preventable diseases, compared to the current immunization strategies? | Delivery | 0.92 | 0.85 | 0.88 | 0.85 | 0.85 | **86.86%** | **0.79** |
| 11 | What is the status of coverage, access, equity, demand and acceptability for immunization of children in Afghanistan after the regime change in August 2021? | Description | 0.87 | 0.88 | 0.88 | 0.85 | 0.84 | **86.53%** | **0.77** |
| 12 | What is the current state of maternal, newborn, and child morbidity and mortality in Afghanistan, especially in rural areas? | Description | 0.85 | 0.87 | 0.87 | 0.89 | 0.84 | **86.39%** | **0.80** |
| 13 | What are current feasible and sustainable options for maintaining and improving quality, accessibility and equity in maternal, newborn and child health service provision, especially in rural areas of Afghanistan? | Delivery | 0.84 | 0.83 | 0.90 | 0.91 | 0.84 | **86.39%** | **0.80** |
| 14 | What is the role of midwives in supporting perinatal mental health? | Delivery | 0.88 | 0.84 | 0.85 | 0.89 | 0.83 | **85.69%** | **0.76** |
| 15 | What intervention models can be used to improve equity in maternal and child health and nutrition in light of vast discrepancies in stunting prevalence and vaccination coverage in different provinces? | Delivery | 0.86 | 0.85 | 0.86 | 0.86 | 0.84 | **85.54%** | **0.75** |
| 16 | What evidence-based maternal health interventions can be implemented for women who cannot access a health facility? | Development | 0.81 | 0.87 | 0.88 | 0.84 | 0.88 | **85.52%** | **0.78** |
| 17 | What are feasible, adaptable and sustainable strategies for conducting and improving mortality reporting and surveillance at national and sub-national levels for maternal, perinatal, stillbirth, newborn and child deaths in Afghanistan? | Delivery | 0.86 | 0.84 | 0.88 | 0.86 | 0.84 | **85.39%** | **0.76** |
| 18 | What are feasible, effective and scalable strategies for integration of mental health and psychosocial support in MNCH programs? | Delivery | 0.82 | 0.87 | 0.87 | 0.84 | 0.83 | **84.60%** | **0.74** |
| 19 | What is the current maternal mortality ratio and what are the primary contributors to maternal mortality in Afghanistan? | Description | 0.80 | 0.84 | 0.88 | 0.86 | 0.85 | **84.45%** | **0.77** |
| 20 | What is the role of non-governmental organizations and civil society organizations in facilitating and delivering women's basic health services in the context of severe social restrictions? | Delivery | 0.88 | 0.79 | 0.84 | 0.87 | 0.78 | **83.09%** | **0.75** |
| 21 | What health services do women, including pregnant women, adolescent girls, children, and other vulnerable populations use, need and want, at national and subnational levels in Afghanistan? | Description | 0.81 | 0.79 | 0.83 | 0.83 | 0.83 | **81.68%** | **0.72** |
| 22 | How can maternal, newborn and child health be improved in a context where policies have severely limited girls' and women's education? | Development | 0.78 | 0.82 | 0.88 | 0.78 | 0.83 | **81.68%** | **0.72** |
| 23 | What are feasible solutions for meeting the critical health needs for women, including pregnant women, adolescent girls, children, and other vulnerable populations at national and subnational levels in Afghanistan? | Development | 0.76 | 0.81 | 0.84 | 0.81 | 0.82 | **80.81%** | **0.69** |
| 24 | What is the effectiveness of measles vaccination, including the seroconversion rate of measles-containing-vaccine first dose (MCV1) and measles-containing-vaccine second dose (MCV2) in Afghanistan? | Description | 0.80 | 0.84 | 0.75 | 0.81 | 0.79 | **79.77%** | **0.72** |
| 25 | How can child immunization services in Afghanistan be sustained after GAVI funding (for equitable and sustainable vaccination of children in low- and middle-income countries) is phased out? | Delivery | 0.83 | 0.80 | 0.78 | 0.78 | 0.80 | **79.64%** | **0.68** |
| 26 | What are immediate actions that would maintain the gains achieved in maternal and child health outcomes in Afghanistan in the past decade? | Development | 0.78 | 0.81 | 0.81 | 0.78 | 0.78 | **79.25%** | **0.68** |
| 27 | What are the ways in which children's health can be appropriately prioritized in Afghanistan? | Delivery | 0.79 | 0.78 | 0.77 | 0.78 | 0.78 | **77.86%** | **0.70** |
| 28 | How has the regime-change in August 2021 impacted maternal, newborn and child mortality and burden of disease, as well as health service (e.g., antenatal care, postnatal care, skilled birth attendance) provision, access and utilization nationally and subnationally in Afghanistan? | Description | 0.73 | 0.76 | 0.82 | 0.75 | 0.75 | **76.37%** | **0.65** |
| 29 | How do facility-based maternal and newborn mortality estimates compare to community-based mortality estimates, when considering the proportion of home birth and attendants, within and across districts in Afghanistan? | Description | 0.75 | 0.75 | 0.76 | 0.79 | 0.72 | **75.37%** | **0.68** |
| 30 | What are the ethical implications of studying maternal, newborn and child health in a context of severe social restrictions on girls and women? | Description | 0.66 | 0.71 | 0.80 | 0.76 | 0.71 | **72.93%** | **0.59** |

## **Table 4.** Overall Ranking, Research Question, 4D’s Domain, Intermediate Research Priority Scores, Overall Research Priority Scores, and Average Expert Agreement for all SRH research questions.

| **Ranking** | **Research Question** | **Domain** | **Feasibility** | **Effectiveness** | **Equity** | **Answerability** | **Disease Burden Reduction** | **Overall RPS** | **AEA** |
| --- | --- | --- | --- | --- | --- | --- | --- | --- | --- |
| 1 | What are feasible demand-side and supply-side options for promoting adequate birth spacing in Afghanistan? | Development | 0.74 | 0.85 | 0.84 | 0.87 | 0.83 | **82.49%** | **0.70** |
| 2 | What are the perceptions of men in Afghanistan toward family planning methods and what factors predict their contraception usage? | Description | 0.79 | 0.82 | 0.81 | 0.79 | 0.77 | **79.64%** | **0.68** |
| 3 | What is the extent of women's access to sexual and reproductive health services in Afghanistan, including maternity and well woman and family planning services; and how does this access vary among women of reproductive age living in urban, peri-urban, and rural areas of the country? | Description | 0.75 | 0.84 | 0.83 | 0.76 | 0.77 | **79.05%** | **0.70** |
| 4 | How does the current state of family planning services in Afghanistan impact maternal, newborn, child, and reproductive health in the country? | Description | 0.70 | 0.77 | 0.84 | 0.78 | 0.77 | **77.30%** | **0.64** |
| 5 | What is the disease burden of reproductive tract infections among women in Afghanistan? | Description | 0.73 | 0.79 | 0.83 | 0.76 | 0.73 | **76.73%** | **0.64** |
| 6 | What is the status of menstrual hygiene management among women and girls in Afghanistan? | Description | 0.69 | 0.81 | 0.84 | 0.75 | 0.70 | **75.69%** | **0.63** |
| 7 | Several barriers to contraception uptake have been identified in the literature. What are the current factors that have led to the stagnation of the modern contraceptive prevalence rate (mCPR) in Afghanistan since 2010, despite the country being positioned for rapid growth of mCPR, and what are the possible interactions and magnitudes of effect of the identified barriers to contraception uptake? | Description | 0.59 | 0.75 | 0.76 | 0.74 | 0.69 | **70.41%** | **0.57** |
| 8 | What are acceptable options for reducing child marriage and promoting contraceptive use in Afghanistan under the current regime? | Development | 0.40 | 0.74 | 0.72 | 0.65 | 0.74 | **65.05%** | **0.59** |
| 9 | What are common causes and contributing factors to infertility in Afghanistan? | Description | 0.55 | 0.63 | 0.61 | 0.61 | 0.52 | **58.11%** | **0.45** |

## **Table 5.** Overall Ranking, Research Question, 4D’s Domain, Intermediate Research Priority Scores, Overall Research Priority Scores, and Average Expert Agreement for all nutrition research questions.

| **Ranking** | **Research Question** | **Domain** | **Feasibility** | **Effectiveness** | **Equity** | **Answerability** | **Disease Burden Reduction** | **Overall RPS** | **AEA** |
| --- | --- | --- | --- | --- | --- | --- | --- | --- | --- |
| 1 | What are potential interventions that are locally adaptable and scalable for preventing and managing the most common causes of child morbidity and mortality in Afghanistan, including acute respiratory infections, diarrheal illnesses, and severe/moderate acute malnutrition? | Development | 0.92 | 0.92 | 0.90 | 0.90 | 0.87 | **90.15%** | **0.83** |
| 2 | What are local sources of nutrition in Afghanistan to prevent child malnutrition and what are strategies to promote these sources? | Development | 0.93 | 0.90 | 0.85 | 0.89 | 0.89 | **89.27%** | **0.82** |
| 3 | What is the association between maternal knowledge, attitudes, and practices on adequate complementary feeding and malnutrition in children in Afghanistan? | Description | 0.90 | 0.87 | 0.88 | 0.91 | 0.86 | **88.21%** | **0.82** |
| 4 | What is the updated prevalence of micronutrient deficiencies (Vitamin A, iron, zinc, etc) among girls, women of reproductive age (15-49 years) and children under age 5 years at the national, province and district level in Afghanistan? | Description | 0.81 | 0.92 | 0.92 | 0.89 | 0.87 | **87.97%** | **0.80** |
| 5 | What are the most effective communication strategies for increasing awareness of Maternal, Infant and Young Child Nutrition (MIYCN) among the Afghan population? | Delivery | 0.90 | 0.87 | 0.87 | 0.90 | 0.84 | **87.41%** | **0.80** |
| 6 | What is the impact of community-level interventions for improving nutrition outcomes, including community-based growth monitoring and promotion and community level management of acute malnutrition? | Delivery | 0.84 | 0.91 | 0.88 | 0.86 | 0.87 | **87.35%** | **0.78** |
| 7 | What is the role of malnutrition in the health crisis women and children in Afghanistan are currently facing, and what are feasible, effective and scalable strategies for integrated MNCH and nutrition services in Afghanistan (e.g. management of mothers and infants at risk of moderate to severe malnutrition)? | Description | 0.88 | 0.90 | 0.87 | 0.86 | 0.85 | **87.22%** | **0.78** |
| 8 | What nutritional interventions and resources are currently in place to address iron-deficiency anemia among women of childbearing age in Afghanistan and to what extent are these initiatives addressing the issue? | Delivery | 0.88 | 0.87 | 0.86 | 0.90 | 0.81 | **86.19%** | **0.77** |
| 9 | What strategies can be employed to identify vulnerabilities in food and nutrition security and effectively respond and provide emergency food assistance in Afghanistan? | Delivery | 0.90 | 0.86 | 0.85 | 0.84 | 0.83 | **85.63%** | **0.76** |
| 10 | What is the nutritional status (including stunting, wasting, and underweight) of children under the age of five in Afghanistan after the regime change? | Description | 0.84 | 0.84 | 0.88 | 0.87 | 0.84 | **85.21%** | **0.78** |
| 11 | What are the prevailing infant and young child feeding (IYCF) practices among children under 24 months in Badakhshan province, Afghanistan, and what insights can be drawn regarding the effectiveness of current nutrition interventions and recommendations for new interventions and scale-up strategies in the region? | Description | 0.89 | 0.83 | 0.85 | 0.90 | 0.77 | **84.85%** | **0.75** |
| 12 | What is the prevalence of anemia among school children in Afghanistan, and how does it affect their academic performance? | Description | 0.79 | 0.83 | 0.85 | 0.84 | 0.82 | **82.55%** | **0.77** |
| 13 | What are the prevalence and associated risk factors for anemia among pregnant women and women of childbearing age in Kandahar City as compared to Afghanistan overall? | Description | 0.79 | 0.83 | 0.77 | 0.87 | 0.78 | **80.81%** | **0.74** |
| 14 | What are possible school-based nutrition programme types/models that can reduce adolescent malnutrition, and school dropout in Afghanistan? | Development | 0.78 | 0.79 | 0.78 | 0.82 | 0.75 | **78.38%** | **0.70** |
| 15 | Is there a significant impact of Vitamin D levels on the growth of children residing in apartment-style housing in Afghanistan? | Description | 0.77 | 0.73 | 0.74 | 0.73 | 0.63 | **72.14%** | **0.62** |
| 16 | Following the regime change, how has the food insecurity situation affected child marriage in Afghanistan? | Description | 0.59 | 0.62 | 0.73 | 0.68 | 0.63 | **65.05%** | **0.52** |
| 17 | How has the freezing of $9 billion foreign reserves after the regime change impacted the current health/malnutrition crisis for women and children in Afghanistan? | Description | 0.56 | 0.52 | 0.55 | 0.58 | 0.51 | **54.20%** | **0.40** |
